# Hypersensitivity to non-β-lactam antibiotics 

**DOI:** 10.5414/ALX02311E

**Published:** 2022-01-24

**Authors:** Hans F. Merk, David R. Bickers

**Affiliations:** 1 Department of Dermatology and Allergology, RWTH Aachen University, Aachen, Germany, and; 2 Department of Dermatology, Columbia University Irving Medical Center, New York, NY, USA

**Keywords:** non-β-Lactam-antibiotics, photosensitivity, MRGPRX2, lymphocyte transformation test, allergy

## Abstract

Most allergic reactions to antibiotics are caused by β-lactam antibiotics; however non-β-lactam antibiotics are also capable of causing both immediate allergic reactions as well as late-type reactions to these drugs. This is especially true for fluoroquinolones and sulfonamides. Of these, the combination of sulfamethoxazole with trimethoprim (Cotrimoxazol, e.g., Bactrim) is most important. However, there are certain types of reactions to non-β-lactam antibiotics that are not associated with β-lactam antibiotics. These include photosensitivity to sulfonamides, tetracyclines, and fluoroquinolones as well as different patterns of drug metabolism and associations with HLA alleles that may influence their prevalence. This review is focused on recent findings regarding the pathogenesis of allergic reactions to non-β-lactam antibiotics.

## Introduction 

Compared to other drug families, antibiotics frequently trigger allergic drug reactions, and in particular β-lactam antibiotics are the major cause of these reactions [3]. This class of antibiotics is widely prescribed for the treatment of numerous infectious diseases. However, non-β-lactam antibiotics can also trigger immediate allergic reactions ranging from urticaria to anaphylactic shock as well as delayed-type reactions [21, 36]. Immediate-type allergic reactions are mediated by specific IgE immunoglobulins in most – if not all – cases typically occurring within seconds to minutes following oral, parenteral, or even topical drug exposure. Most often, they occur within 1 hour after exposure. Sometimes they can develop after several hours – but rarely after more than 6 hours. One explanation for the latter may be delayed drug absorption. In contrast, delayed-type reactions can occur days after drug administration. More than 90% of these cutaneous reactions appear as maculopapular or morbilliform eruptions, are not life-threatening, and usually resolve spontaneously with or without topical therapy once the offending drug has been withdrawn. However, 5 – 10% of these reactions may result in more severe cutaneous adverse reactions (SCARs) such as bullous drug eruptions as well as fixed or multiple fixed drug eruptions, Stevens-Johnson Syndrome (SJS), toxic epidermal necrolysis (TEN) but also acute generalized exanthematous pustulosis (AGEP) and drug rash with eosinophilia and systemic symptoms (DRESS) [3]. Among these, TEN is the most severe type of delayed drug reaction, but fortunately it is quite rare with an incidence of 1 – 2/million people/year although up to one-third of these patients will not survive. The medical management of these allergic reactions does not differ significantly from corresponding reactions to β-lactam antibiotics. Diagnostic skin testing methods for immediate reactions include both prick tests and intracutaneous tests also known as intradermal tests, whereas in late reactions, patch testing is the preferred diagnostic procedure. The diagnosis of immediate reactions typically consists of obtaining a careful medication history along with the tests described above. Additionally, the determination of specific IgE antibodies and the basophil activation test can be used. The latter is a functional assay that measures the degree of degranulation following stimulation with allergens or controls by flow cytometry. In selected cases, a drug provocation test may be appropriate [26]. For delayed hypersensitivity reactions, the diagnostic methods also include a careful history along with the patch or intracutaneous test with delayed reading as well as in vitro tests including the lymphocyte transformation test or the ELISpot assay and, in selected cases, a drug provocation test [3, 31]. These procedures are performed only in patients with maculopapular exanthems and are in parts contraindicated in patients with SCARs. However, it is important to remember that doses of suspected drugs used for provocation tests are much less well-defined than such testing with β-lactam antibiotics, in particular with regard to penicillin allergy [4, 22, 29]. 

As mentioned above, there are unique reactions to non-β-lactam antibiotics that are not seen in patients with β-lactam antibiotics, including phototoxic or photoallergic reactions to various antibiotics including sulfonamides, tetracyclines, and fluoroquinolones [18]. The pathophysiologic mechanism of allergic drug reactions to sulfonamides involves metabolic activation by cytochrome P450 isoenzymes (CYP) into highly reactive sensitizing derivatives that can bind to macromolecules [27, 32]. In contrast, β-lactam antibiotics are for the most part not substrates for CYPs or other oxidizing enzymes [27]. Anaphylactic reactions to fluoroquinolones can be triggered by IgE-dependent pathways and by direct activation of mast cells [8, 25]. 

## Photosensitivity 

Unlike β-lactam antibiotics, sulfonamides, fluoroquinolones, and tetracycline antibiotics can trigger both phototoxic and photoallergic reactions [18]. Clinically, these reactions are characterized by the acute onset of erythema and/or an eczematous dermatitis after skin exposure to solar ultraviolet radiation (UVR) on sun-exposed body areas. Therefore, mainly the “sun terraces” of the skin including the back of the hands, the central face, the lower lip, the ears, and the balding scalp are typically affected in these reactions. In contrast, certain anatomic areas such as the upper eyelids are typically spared due to the photoprotective effects of the retraction of the open eyelids. Currently, it is the fluoroquinolones that are the major non-β-lactam antibiotics that cause these types of adverse reactions [18] . 

**Figure 1:**
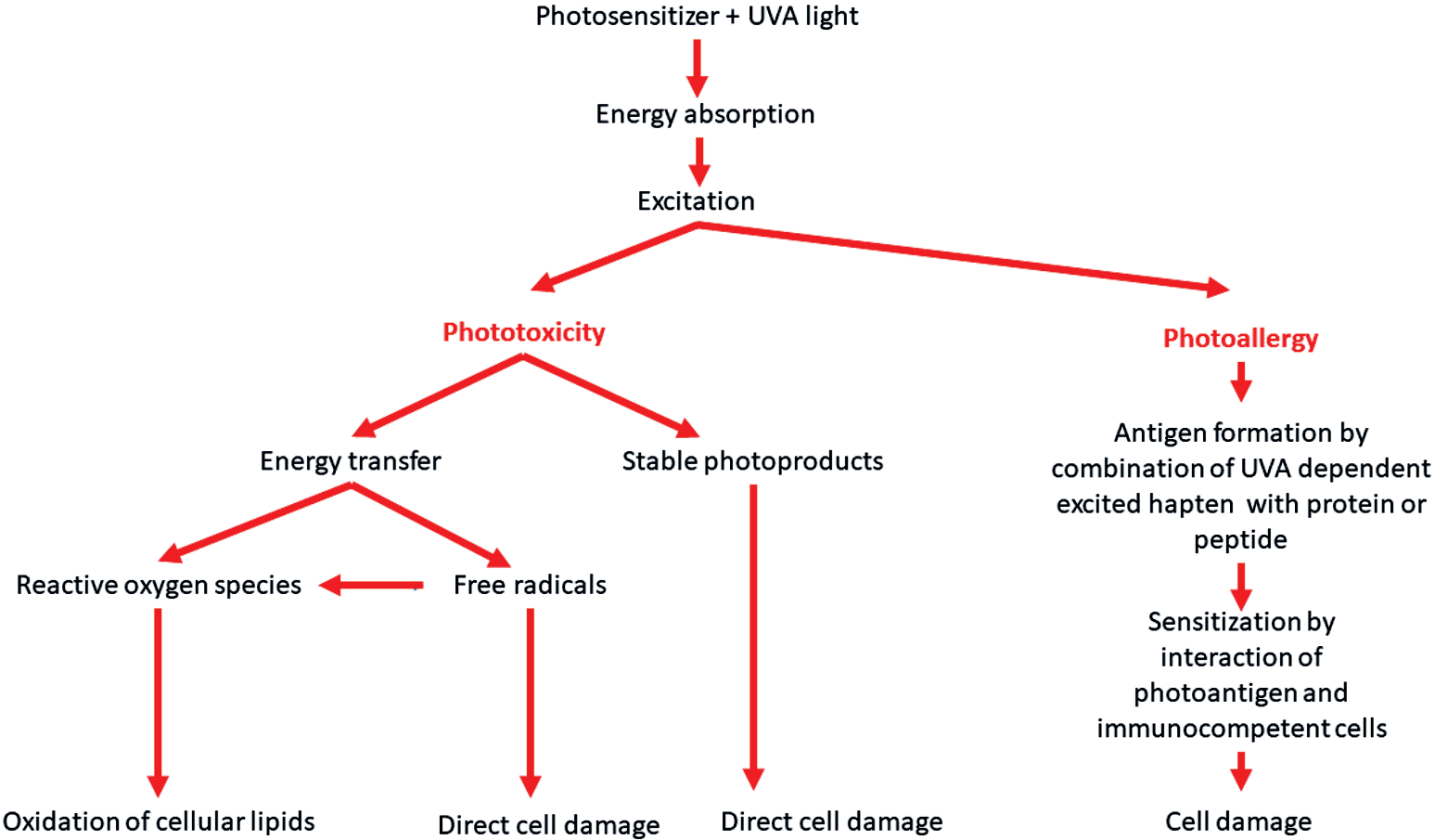
Hypothetical mechanisms of photosensitivity reactions including phototoxic and photoallergic reactions [12, 14].

The mechanisms involved in these reactions are complex [11]. It is the combination of administering a drug that has an absorption spectrum between 320 and 400 nm (the ultraviolet A (UVA) range) followed by exposure to solar UVR that triggers the reaction [15, 17]. Photon absorption by the drug drives the generation of reactive oxygen species (ROS) and/or highly reactive drug metabolites (Figure 1). These chemically reactive drugs or their metabolites can bind to macromolecules to form a complete allergen after interacting with immunocompetent cells such as antigen-presenting dendritic cells and T lymphocytes and in the process induce a photoallergic reaction that is typically an eczematous dermatitis. Both ROS and the photoexcited drug may also have a non-immunogenic direct cytotoxic effect that induces a phototoxic reaction that manifests as erythema resembling an exaggerated sunburn [10]. The ultimate risk of developing these reactions is dependent upon drug concentration in the skin and the intensity of exposure to solar UVR. At the same time, the skin possesses a multitude of enzymatic and non-enzymatic photoprotective mechanisms to minimize phototoxic and photoallergic reactions. The enzymatic antioxidants include catalases, glutathione peroxidase, superoxide dismutase, and reductases, among others [14]. The non-enzymatic photoprotective agents include antioxidants such as glutathione, α-tocopherol, ascorbate and β-carotene, among others. 

The suspicion of a photosensitivity is based upon the patient’s history of exposure. The standard procedures for confirming the diagnosis are known as the minimum erythema dose (MED) test and the photopatch test (PPT), and a detailed description of these procedures has recently been published [7]. 

Briefly, on day 1, photo tests are performed using artificial light sources that emit UVB and UVA radiation to determine the MED to UVB and UVA, and on that day up to 24 photoallergens (Chemotechnique Diagnostics AB, Malmo, Sweden), often supplemented with a baseline series and including any products that the patient may be using, are applied in duplicate to the back of the patient with a standardized technique using Finn Chambers (Epitest Ltd. Oy, Tuusula, Finland) on Scanpor tape (Norgesplaster Alpharma AS, Vennesla, Norway). On day 2 or day 3, the patches are removed and one of the duplicate sets are irradiated with UVA (10 J/cm^2^). If the UVA MED was less than 10 J/cm^2^, a dose of at least one J/cm^2^less than the MED is used. The remaining set of allergens acts as non-irradiated control. Two readings of the test sites are performed in each patient, on day 3 or 4 and one of days 5 – 10. Patch test reactions are scored as +/− (weak/doubtful/macular erythema), + (mild), ++ (strong), or +++ (very strong). If both the non-irradiated and irradiated sites show equally positive reactions, this suggests the diagnosis of allergic contact dermatitis; if positive reactions occur only at the irradiated sites, a diagnosis of photoallergic contact dermatitis is established; if both sites show positive reactions but the irradiated site is more intensely positive, a diagnosis of photo-aggravated contact dermatitis is likely. Positive reactions are considered to be clinically relevant if the patient has had known definite, probable, possible, or past contact with the suspected allergen and the timing of exposure coincides with the development of photodermatitis. 

## Sulfonamides 

Allergic reactions to sulfonamides occur in ~ 2 – 4% of patients treated with these drugs. For unknown reasons, patients with HIV have a 10-fold increased risk of these reactions [29]. The clinical presentation in these patients includes maculopapular rash and/or SCARs, whereas anaphylactic reactions are quite rare. Currently, the most common source of exposure to sulfonamides comes from the administration of Cotrimoxazole which contains the combination of sulfamethoxazole (SMX) and trimethoprim e.g., Bactrim (Roche, Basel, Switzerland). Unfortunately, in most cases prick/intracutaneous tests and patch tests are not sufficiently sensitive to detect allergic reactions to either sulfonamides or trimethoprim. In the rare case of immediate-type reactions, prick and intracutaneous testing is recommended. It is of interest that certain HLA associations in some ethnic groups may influence susceptibility to SMX reactions as well as to dapsone, a sulfone derivative (Table 1) [28]. 

Sulfonamides including SMX are metabolized by xenobiotic-metabolizing enzymes including CYP450 isoenzymes. SMX is converted by cytochrome P450 (CYP) 2C9 or peroxidases to SMX-hydroxylamine, which spontaneously forms nitroso-SMX with oxygen, and nitroso-SMX in turn reacts with SMX-hydroxylamine to generate azo- and azoxydimers; furthermore nitroso-SMX is converted to the more stable NO-SMX. [9]. 

There is convincing evidence from in vitro and in vivo animal studies that this metabolic pathway plays an important role in both sensitizing and eliciting allergic reactions to these drugs, in particular SMX [35]. We have conducted studies using lesional T lymphocyte clones obtained from the involved skin of a patient suffering from SJS to SMX. These lymphocyte clones specifically recognized SMX; however, they reacted only after incubation of SMX together with liver microsomes, which we have shown to contain CYP isoenzymes [13]. The significance of the SMX oxidation to NO-SMX as a prerequisite for driving the allergic reactions was also demonstrated by immunohistological studies using specific antibodies directed against NO-SMX. Co-staining with NO-SMX antibodies and MHC I-binding antibodies was detected on keratinocytes, suggesting that this reactive metabolite is capable of binding to immunologically critical macromolecules [30]. 

More recently, using an in vitro priming assay consisting of T lymphocytes and antigen-presenting cells (APCs), sensitization to SMX was shown to be due to NO-SMX. This is produced by a CYP2C9-dependent metabolic activation [10]. 

As mentioned above, human exposure to SMX occurs most frequently in patients receiving Cotrimoxazole, e.g., Bactrim. In addition, trimethoprim itself can also cause allergic reactions. Recent studies in an animal model of trimethoprim reactions showed that hydroxylated trimethoprim and its reactive sulfated metabolites can bind to skin proteins suggesting their involvement in an allergic reaction [5]. 

Cross-reactions between sulfonamides and drugs such as dapsone or non-antimicrobial drugs with a sulfonamide-like structure such as furosemide or celecoxib have not been documented [16]. However, T-cell clones have recently been isolated from HLA-B*13:01-positive patients. This HLA allele is associated with an increased risk of SCARs to SMX and dapsone in patients from Thailand (Table 1). Individual clones that reacted to SMX also reacted to nitroso-dapsone [28]. 

## Antibiotics as ligands of MRGPRX2 

Mas-related G-protein-coupled receptor member X2 (MRGPRX2) plays a role in the interaction between mast cells and the microbiome. It is expressed predominantly by mast cells, but also by basophils and eosinophils and can be activated by binding to various drug ligands including antibiotics [20, 34]. This mast cell-associated activation can lead to IgE-independent anaphylactic reactions. Fluoroquinolones are also known to bind to MRGPRX2. The incidence of fluoroquinolone-induced anaphylaxis was described as 0.2 per 1 million days of treatment [2]. Fluoroquinolones are also capable of inducing IgE-mediated allergic reactions as well as pseudo-allergic reactions by activation of mast cells after binding to MRGPRX2 [1, 23, 24]. In one review of severe hypersensitivity reactions to non-β-lactam antibiotics, fluoroquinolones were involved in at least half of the anaphylactic reactions [32]. The development of anaphylaxis immediately after an initial dose or within the first 3 days of administration suggests that not all reactions require prior sensitization and are therefore unlikely to be IgE-mediated [33]. Pseudo-allergic anaphylactic reactions to fluoroquinolones may also be dependent on their pharmacokinetics and their binding affinity to MRGPRX2 perhaps related to delayed drug elimination [25]. Among the fluoroquinolones that bind to MRGPRX2, ciprofloxacin has the highest binding affinity with an EC_50_ value of 6.8 μg/mL, whereas for levofloxacin it is 22.7 μg/mL [25]. The basophil activation test may also be helpful as it would yield positive results only in the case of IgE-dependent reactions [8]. Mast cell activation by fluoroquinolones may also be associated with other adverse clinical effects of these antibiotics such as arthralgias, myalgias, or joint damage, as they accumulate in cartilage tissue and then trigger local mast cell-dependent inflammatory responses [25]. 

Vancomycin is a glycopeptide antibiotic capable of inducing Red man syndrome due to its binding to MRGPRX2 that can also trigger anaphylactic reactions [25]. The method of administration (oral or IV) as well as the speed of the infusion may be risk factors. SCARs, in particular vancomycin-associated DRESS, have been described in association with HLA-A*32:01 [19]. 

## Macrolide antibiotics 

**Figure 2:**
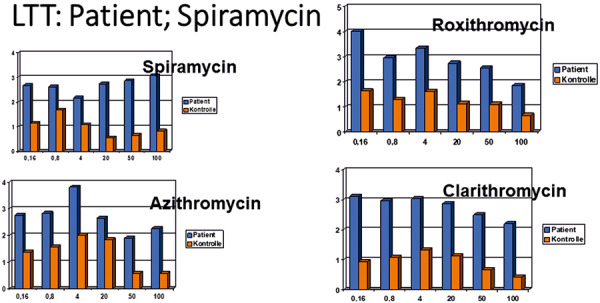
A lymphocyte transformation test was performed on a patient who developed a spiramycin-dependent drug rash while working at a chemical plant where this drug was manufactured. Cross-reactivity was observed with azithromycin, roxithromycin, and clarithromycin. SI = stimulationindex. PHA: 8.74 (control: 8.52); TT: 3.09 (control: 25.65); concentration of antibiotics in µg/mL.

Allergic reactions to this group of non-β-lactam antibiotics that include erythromycin, troleandomycin, clarithromycin, spiramycin, and josamycin, are rare compared to the other antibiotics discussed above. Diagnostic skin testing procedures are considered to be less sensitive in assessing reactions to these antibiotics [29]. In our own observation of a morbilliform drug rash to spiramycin that was acquired occupationally in a manufacturing plant, we were able to confirm broad cross-reactivity across various macrolide antibiotics (Figure 2). 

## Clindamycin 

**Figure 3:**
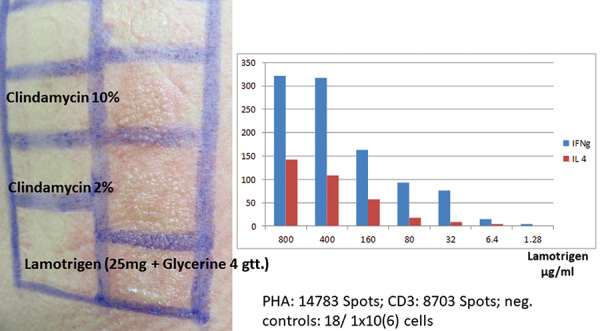
Example of a positive ELISpot assay with IFN-γ release after incubation with lamotrigine in a patient diagnosed with DRESS caused by lamotrigine together with clindamycin. There was no activation in the ELISpot assay to clindamycin; however, the patch test was for clindamycin as well as for lamotrigine. The patient was taking both medications when DRESS appeared.

Clindamycin is frequently associated with delayed-type reactions. Clindamycin can induce significant positive patch test reactions. As an example, in Figure 3 a patient was shown to be suffering from lamotrigine-associated DRESS based on positive reactions in the ELISpot assay and with patch testing; however, she also showed a positive patch test reaction to clindamycin, but the ELISpot assay was negative (Figure 3). The patient had been treated with both medications prior to developing the DRESS reaction. 

## Conclusion 

In summary, this review demonstrates that in comparison to β-lactam antibiotics, skin and in vitro tests for reactions to non-β-lactam antibiotics have not been so rigorously tested in verifying their involvement in affected patients. Indeed, they are mostly based on published individual case reports. Nonetheless, these procedures may sometimes be helpful in confirming a suspected diagnosis, in particular in severe allergic drug reactions such as DRESS, for which provocation testing is contraindicated. 

## Funding 

This study was supported in part by the NIH/National Institute of Arthritis and Musculoskeletal and Skin Diseases (NIAMS) funded Skin Disease Research Center in the Department of Dermatology (P30AR69632) at Columbia University (D.B.). 

## Conflict of interest 

No conflict of interest (H.M., D.R.B.). 

**Table 1.** HLA association with various phenotypes of drug hypersensitivity to non-β-lactam antibiotics in different populations [6].

Associated drug	HLA allele	Drug allergy	Ethnicity
Co-trimoxasole	B*15:02, C*06:02, C*08:01 B*13:01	SJS/TEN	Thai
Sulfa-methoxazole	B*38:02	SJS/TEN	European
Dapsone	B*13:01	DRESS	Han-chinese, Thai

## References

[1] Aranda A Mayorga C Ariza A Doña I Rosado A Blanca-Lopez N Andreu I Torres MJ In vitro evaluation of IgE-mediated hypersensitivity reactions to quinolones. Allergy. 2011; 66: 247–254. 20722637 10.1111/j.1398-9995.2010.02460.x

[2] Blayac JP Hillaire-Buys D Pinzani V Fluoroquinolones and anaphylaxis. Therapie. 1996; 51: 417–418. 8953820

[3] Blumenthal KG Peter JG Trubiano JA Phillips EJ Antibiotic allergy. Lancet. 2019; 393: 183–198. 30558872 10.1016/S0140-6736(18)32218-9 PMC6563335

[4] Brockow K Garvey LH Aberer W Atanaskovic-Markovic M Barbaud A Bilo MB Bircher A Blanca M Bonadonna B Campi P Castro E Cernadas JR Chiriac AM Demoly P Grosber M Gooi J Lombardo C Mertes PM Mosbech H Nasser S Skin test concentrations for systemically administered drugs – an ENDA/EAACI Drug Allergy Interest Group position paper. Allergy. 2013; 68: 702–712. 23617635 10.1111/all.12142

[5] Cao Y Bairam A Jee A Liu M Uetrecht J Investigating the Mechanism of Trimethoprim-Induced Skin Rash and Liver Injury. Toxicol Sci. 2021; 180: 17–25. 33394045 10.1093/toxsci/kfaa182 PMC7916736

[6] Chen CB Abe R Pan RY Wang CW Hung SI Tsai YG Chung WH An Updated Review of the Molecular Mechanisms in Drug Hypersensitivity. J Immunol Res. 2018; 2018:6431694. 29651444 10.1155/2018/6431694 PMC5830968

[7] DeLeo VA Adler BL Warshaw EM Maibach HI Belsito DV Fowler JF Fransway AF Marks JG Mathias CGT Pratt MD Rietschel RL Storrs FJ Zug KA Sasseville D Photopatch test results of the North American contact dermatitis group, 1999 – 2009. Photodermatol Photoimmunol Photomed. 2021;epub ahead of print. 10.1111/phpp.12742 34651348

[8] Doña I Pérez-Sánchez N Salas M Barrionuevo E Ruiz-San Francisco A Hernández Fernández de Rojas D Martí-Garrido J Andreu-Ros I López-Salgueiro R Moreno E Torres MJ Clinical Characterization and Diagnostic Approaches for Patients Reporting Hypersensitivity Reactions to Quinolones. J Allergy Clin Immunol Pract. 2020; 8: 2707–2714.e2. 32376487 10.1016/j.jaip.2020.04.051

[9] Earnshaw CJ Pecaric-Petkovic T Park BK Naisbitt DJ 2014;104:137-163. 10.1007/978-3-0348-0726-5_10 24214623

[10] Faulkner L Gibson A Sullivan A Tailor A Usui T Alfirevic A Pirmohamed M Naisbitt DJ Kevin Park B Detection of Primary T Cell Responses to Drugs and Chemicals in HLA-Typed Volunteers: Implications for the Prediction of Drug Immunogenicity. Toxicol Sci. 2016; 154: 416–429. 27637899 10.1093/toxsci/kfw177

[11] Harber LC Current status of mammalian and human models for predicting drug photosensitivity. J Invest Dermatol. 1981; 77: 65–70. 7252259 10.1111/1523-1747.ep12479249

[12] Harber LC Bickers DR Photosensitivity Diseases. Philadelphia:W.B. Saunders; 1981.

[13] Hertl M Jugert F Merk HF CD8+ dermal T cells from a sulphamethoxazole-induced bullous exanthem proliferate in response to drug-modified liver microsomes. Br J Dermatol. 1995; 132: 215–220. 7534104 10.1111/j.1365-2133.1995.tb05016.x

[14] Hofmann GA Weber B Drug-induced photosensitivity: culprit drugs, potential mechanisms and clinical consequences. J Dtsch Dermatol Ges. 2021; 19: 19–29. 10.1111/ddg.14314 PMC7898394 33491908

[15] Ibbotson SH Shedding light on drug photosensitivity reactions. Br J Dermatol. 2017; 176: 850–851. 28418140 10.1111/bjd.15449

[16] Khan DA Knowles SR Shear NH Sulfonamide Hypersensitivity: Fact and Fiction. J Allergy Clin Immunol Pract. 2019; 7: 2116–2123. 31495421 10.1016/j.jaip.2019.05.034

[17] Khandpur S Porter RM Boulton SJ Anstey A Drug-induced photosensitivity: new insights into pathomechanisms and clinical variation through basic and applied science. Br J Dermatol. 2017; 176: 902–909. 27510322 10.1111/bjd.14935

[18] Kim WB Shelley AJ Novice K Joo J Lim HW Glassman SJ Drug-induced phototoxicity: A systematic review. J Am Acad Dermatol. 2018; 79: 1069–1075. 30003982 10.1016/j.jaad.2018.06.061

[19] Konvinse KC Trubiano JA Pavlos R James I Shaffer CM Bejan CA Schutte RJ Ostrov DA Pilkinton MA Rosenbach M Zwerner JP Williams KB Bourke J Martinez P Rwandamuriye F Chopra A Watson M Redwood AJ White KD Mallal SA HLA-A*32:01 is strongly associated with vancomycin-induced drug reaction with eosinophilia and systemic symptoms. J Allergy Clin Immunol. 2019; 144: 183–192. 30776417 10.1016/j.jaci.2019.01.045 PMC6612297

[20] Kühn H Kolkhir P Babina M Düll M Frischbutter S Fok JS Jiao Q Metz M Scheffel J Wolf K Kremer AE Maurer M Mas-related G protein-coupled receptor X2 and its activators in dermatologic allergies. J Allergy Clin Immunol. 2021; 147: 456–469. 33071069 10.1016/j.jaci.2020.08.027

[21] Kuyucu S Mori F Atanaskovic-Markovic M Caubet JC Terreehorst I Gomes E Brockow K Hypersensitivity reactions to non-betalactam antibiotics in children: an extensive review. Pediatr Allergy Immunol. 2014; 25: 534–543. 25201401 10.1111/pai.12273

[22] Macy E Romano A Khan D Practical Management of Antibiotic Hypersensitivity in 2017. J Allergy Clin Immunol Pract. 2017; 5: 577–586. 28365277 10.1016/j.jaip.2017.02.014

[23] Manfredi M Severino M Testi S Macchia D Ermini G Pichler WJ Campi P Detection of specific IgE to quinolones. J Allergy Clin Immunol. 2004; 113: 155–160. 14713922 10.1016/j.jaci.2003.09.035

[24] McNeil BD Pundir P Meeker S Han L Undem BJ Kulka M Dong X Identification of a mast-cell-specific receptor crucial for pseudo-allergic drug reactions. Nature. 2015; 519: 237–241. 25517090 10.1038/nature14022 PMC4359082

[25] McNeil BD MRGPRX2 and Adverse Drug Reactions. Front Immunol. 2021; 12:676354. 34421893 10.3389/fimmu.2021.676354 PMC8377365

[26] Merk HF Saloga J Klimek L Buhl R Mann WJ Knop J Grabbe S 2012; 426–439.

[27] rzneimittelmetabolismus als Risikofaktor kutaner Medikamentenallergie. Allergologie. 2017; 40: 476–482.

[28] Pratoomwun J Thomson P Jaruthamsophon K Tiyasirichokchai R Jinda P Rerkpattanapipat T Tassaneeyakul W Nakkam N Rerknimitr P Klaewsongkram J Srinoulprasert Y Pirmohamed M Naisbitt DJ Sukasem C Characterization of T-Cell Responses to SMX and SMX-NO in Co-Trimoxazole Hypersensitivity Patients Expressing HLA-B*13:01. Front Immunol. 2021; 12:658593. 33995375 10.3389/fimmu.2021.658593 PMC8117787

[29] Romano A Warrington R Antibiotic allergy. Immunol Allergy Clin North Am. 2014; 34: 489–506, vii.. 25017674 10.1016/j.iac.2014.03.003

[30] Roychowdhury S Vyas PM Reilly TP Gaspari AA Svensson CK Characterization of the formation and localization of sulfamethoxazole and dapsone-associated drug-protein adducts in human epidermal keratinocytes. J Pharmacol Exp Ther. 2005; 314: 43–52. 15784651 10.1124/jpet.105.086009

[31] Sachs B Fatangare A Sickmann A Glässner A Lymphocyte transformation test: History and current approaches. J Immunol Methods. 2021; 493:113036. 33745950 10.1016/j.jim.2021.113036

[32] Sachs B Fischer-Barth W Merk HF Reporting rates for severe hypersensitivity reactions associated with prescription-only drugs in outpatient treatment in Germany. Pharmacoepidemiol Drug Saf. 2015; 24: 1076–1084. 26285651 10.1002/pds.3857

[33] Sachs B Riegel S Seebeck J Beier R Schichler D Barger A Merk HF Erdmann S Fluoroquinolone-associated anaphylaxis in spontaneous adverse drug reaction reports in Germany: differences in reporting rates between individual fluoroquinolones and occurrence after first-ever use. Drug Saf. 2006; 29: 1087–1100. 17061914 10.2165/00002018-200629110-00008

[34] Subramanian H Gupta K Ali H Roles of Mas-related G protein-coupled receptor X2 on mast cell-mediated host defense, pseudoallergic drug reactions, and chronic inflammatory diseases. J Allergy Clin Immunol. 2016; 138: 700–710. 27448446 10.1016/j.jaci.2016.04.051 PMC5014572

[35] Sullivan A Gibson A Park BK Naisbitt DJ Are drug metabolites able to cause T-cell-mediated hypersensitivity reactions? Expert Opin Drug Metab Toxicol. 2015; 11: 357–368. 25495340 10.1517/17425255.2015.992780

[36] Zambrano G Fernandez T Ameiro B Pinto C Alvarez-Perea A De Barrio M Non-Immediate Skin Reactions Due To Antibiotics JACI. 2014; 274:AB945.

